# Identifying patient-reported and clinician-reported outcome measures to compare providers of arthroscopic rotator cuff repair: insights obtained from a Swiss multicentre cohort study

**DOI:** 10.1136/bmjoq-2026-004516

**Published:** 2026-11-11

**Authors:** Werner Vach, Alexandre Lädermann, Matthias A Zumstein, Gregory Cunningham, Bernhard Jost, Thomas Stojanov, Marcel Jakob, Andreas Marc Mueller, Laurent Audigé, ARCR_Pred Study Group

**Affiliations:** 1 Basel Academy for Quality and Research in Medicine, Basel, Switzerland; 2 Division of Orthopaedics and Trauma Surgery, Department of Surgery, Geneva University Hospitals, Geneva, Switzerland; 3 Division of Orthopaedics and Trauma Surgery, La Tour Hospital, Meyrin, Switzerland; 4 Shoulder, Elbow and Orthopaedics Sport Medicine, Sonnenhof Orthopaedics, Bern, Switzerland; 5 Faculty of Medicine, University of Bern, Bern, Switzerland; 6 Shoulder and Elbow Center, La Colline, Geneva, Switzerland; 7 Clinic for Orthopaedic Surgery and Traumatology of the Musculoskeletal System, Cantonal Hospital of St.Gallen, St. Gallen, Switzerland; 8 Orthopaedic Surgery and Traumatology, University Hospital Basel, Basel, Switzerland; 9 Surgical Outcome Research Center, University Hospital Basel, Basel, Switzerland; 10 Crossklinik, Basel, Switzerland; 11 Faculty of Medicine, University of Basel, Basel, Switzerland; 12 Department of Clinical Research, University of Basel, Basel, Switzerland; 13 Research and Development Department, Schulthess Klinik, Zürich, Switzerland

**Keywords:** Surgery, Healthcare quality improvement, Patient Reported Outcome Measures

## Abstract

**Background:**

Healthcare provider comparisons are getting increasingly popular, including in the field of surgery. Outcome comparisons following arthroscopic rotator cuff repair (ARCR) lack guidance on the choice of outcomes and selection of variables for case-mix adjustment for this specific patient group. This study aims to determine the extent to which various endpoints and approaches to case-mix adjustment are suitable for identifying differences in treatment outcomes among surgeons.

**Methods:**

In a multicentre Swiss-German cohort study, 973 patients from 19 different clinics were followed after ARCR for 24 months using patient-reported outcome measures (PROMs) and for 12 months using clinician-reported outcome measures (CROMs). 73 potential outcome indicators were analysed with respect to their ability to discriminate between clinics or surgeons. In addition, a set of 54 potential factors to be used for case-mix adjustment was screened for selection using five different methodological approaches.

**Results:**

Several indicators based on PROMs or CROMs could be identified, which allow detection of differences in postoperative outcomes between providers as early as 6 months following ARCR. These differences persisted after case-mix adjustment, which resulted in only modest reductions in between-provider variation and limited changes in provider rankings. Often, just a few variables—available in clinical routine or from baseline measurements of PROMs—seem to be sufficient for casemix adjustment.

**Conclusions:**

Provider-dependent outcome differences following ARCR are detectable early after surgery using PROMs or CROMs. These differences can only partly be explained by patient case-mix, suggesting variation in the effectiveness of ARCR care delivery across providers. Hence, both PROMs and CROMs offer the opportunity to inform provider comparisons.

**WHAT IS ALREADY KNOWN ON THIS TOPIC**

- Patient-reported outcomes allow demonstrating outcome differences among healthcare providers in many patient groups. It is unknown whether this also applies to providers of arthroscopic rotator cuff repair.

**WHAT THIS STUDY ADDS**

- Both specific patient-reported outcomes and clinician-reported outcomes allow demonstrating outcome differences between providers of arthroscopic rotator cuff repair. A follow-up period of 6 months or less can be sufficient.

**HOW THIS STUDY MIGHT AFFECT RESEARCH, PRACTICE OR POLICY**

- Stakeholders selecting potential outcome quality indicators for comparing providers of arthroscopic rotator cuff repair may consider specific patient-reported outcomes or clinician-reported outcomes.

## Background

 Outcomes following arthroscopic rotator cuff repair (ARCR) vary substantially between patients in routine clinical practice. While this variability is often attributed to differences in baseline patient characteristics, tear severity or comorbidities, it remains unclear to what extent postoperative recovery may also depend on differences in surgical technique, perioperative management or rehabilitation pathways across providers, that is, care delivery. Systematic provider comparisons allow clarifying this question. However, for this specific patient group, there is a lack of guidance on the choice of outcomes and selection of variables for case-mix adjustment.

Patient-reported outcome measures (PROMs) offer a patient-centred way to compare healthcare providers by focusing on what matters most to patients: pain, function and longer-term well-being. England’s National Health Service pioneered routine PROMs collection in 2009 after selected elective procedures,^1^ and interest has since grown.^2–4^ However, provider comparisons based on PROMs remain challenging to implement in national quality assurance projects, and practical experience with adequate case-mix adjustment is still limited.^5 6^

Implementing outcome-based comparisons requires several methodological choices. PROMs can differ in terms of their type (generic vs disease-specific), their target domain (pain/function/quality of life), and their feasibility for routine large-scale collection. An adequate time point for follow-up has to be chosen, and both follow-up scores or changes from baseline can be used. While PROM-based comparisons have been explored for several surgical domains,^7–10^ they have not yet been established for shoulder surgery. In surgical settings, clinician-reported outcome measures (CROMs) may complement or, in some contexts, serve as an alternative to PROMs, particularly when derived from standardised assessment procedures that limit observer influence (eg, objective strength or range-of-motion assessments). In addition, a key requirement for fair provider comparisons is case-mix adjustment,^11 12^ because patient populations differ across providers in terms of baseline characteristics that influence outcomes. Without adjustment, differences in mean outcomes may reflect differences in patient profiles rather than differences in quality of care. For nationwide implementation, it is often preferable to rely on a parsimonious set of routinely available baseline variables. The ARCR_Pred study^13 14^ provides an opportunity to examine these issues in patients undergoing ARCR, a common procedure for rotator cuff tears and a treatment generally considered cost-effective.^15^

The aim of this paper was to evaluate whether specific outcomes following ARCR vary between providers after accounting for patient case-mix and hence may allow to detect differences in quality of care delivery. All outcomes collected in ARCR_Pred were considered as potential indicators. In addition, it was aimed to identify a parsimonious set of baseline characteristics suitable for case-mix adjustment. This way we aimed at informing the design of a future nationwide quality assurance project in Switzerland and its alignment with the Swiss Orthopaedics Minimal Data Set (SOMDS).^4^

## Material and methods

### Study design and data collection

Both the design of the ARCR-Pred study as well as the distribution of baseline characteristics and of selected outcomes have previously been described in detail.^13 14^ The study included 973 patients and collected data across 19 different clinics. Details regarding the number of surgeons and patients per clinic as well as the participating surgeons are provided in table 1. Clinics will be referred to by randomly chosen letters.

**Table 1:** Distribution of clinic and surgeon characteristics

Clinic characteristics	
Number of surgeons per clinic (n=19)	
Mean (SD)	2.9 (2.3)
Range	1.0–8.0
Number of surgeons per clinic (n=19)	
1	7 (36.8%)
2–4	7 (36.8%)
5–8	5 (26.3%)
Number of patients per clinic (n=19)	
Mean (SD)	51.2 (42.4)
Range	7.0–161.0
Clinic private vs public (n=19)	
Public	11 (57.9%)
Private	8 (42.1%)

Surgeon characteristics	
Number of patients per surgeon (n=55)	
Mean (SD)	17.7 (21.9)
5th–95th percentile	1.0–44.0
Number of patients per clinic (n=55)	
−10	29 (52.7%)
11–25	13 (23.6%)
25+	13 (23.6%)
Surgeon position (n=55)	
Senior consultant	29 (52.7%)
Consultant	26 (47.3%)
Years of training/practice in orthopaedics (n=55)	
1–5 years	3 (5.5%)
>5–10 years	22 (40.0%)
>10–20 years	21 (38.2%)
>20 years	9 (16.4%)
Years of training/practice in shoulder surgery (n=55)	
1–5 years	24 (43.6%)
>5–10 years	12 (21.8%)
>10–20 years	14 (25.5%)
>20 years	5 (9.1%)
Average number of rotator cuff repairs performed annually (n=55)	
1–20	13 (23.6%)
>20–50	14 (25.5%)
>50–100	19 (34.5%)
>100	9 (16.4%)

Patients were asked to complete questionnaires at baseline and 6, 12 and 24 months after surgery. They had the choice between doing it at home online or on paper, or during a study visit on a tablet. The PROMs collected and included in this paper are shown in table 2. The EQ5D5L,^16 17^ the question on quality of sleep, the two Patient-Reported Outcomes Measurement Information System (PROMIS) scales on anxiety and depression,^18 19^ and the question on perception of change in quality of life present generic instruments. The questions on shoulder pain, the Subjective Shoulder Value,^20^ the Oxford Shoulder Score^21 22^ and the two questions on perception of change in pain and function present joint-specific instruments. The PROMs cover all eight dimensions of the OMERACT Core Domain Set for Clinical Trials of Shoulder Disorders.^23^ The response rates at follow-up were 94%, 90% and 88%, respectively.

**Table 2:** PROMs collected during follow up and at baseline

PROM	Question and/or response options
Shoulder pain score last week	Score the pain level you have experienced in your shoulder over the last week. (0–10)
Quality of sleep	How is the quality of your sleep over the past week? (0–10)
Subjective shoulder value	If your own shoulder was completely normal, it would have a value of 100%. What value does your (affected) shoulder currently have? This question refers to the period over the last week. (0–100)
EQ5D5L: mobility	I have no problems in walking about/I have slight problems in walking about/I have moderate problems in walking about/I have severe problems in walking about/I am unable to walk about
EQ5D5L: self-care	I have no problems washing or dressing myself/I have slight problems washing or dressing myself/I have moderate problems washing or dressing myself/I have severe problems washing or dressing myself/I am unable to wash or dress myself
EQ5D5L: usual activities	I have no problems doing my usual activities/I have slight problems doing my usual activities/I have moderate problems doing my usual activities/I have severe problems doing my usual activities/I am unable to do my usual activities
EQ5D5L: pain/discomfort	I have no pain or discomfort/I have slight pain or discomfort/I have moderate pain or discomfort/I have severe pain or discomfort/I have extreme pain or discomfort
EQ5D5L: anxiety/depression	I am not anxious or depressed/I am slightly anxious or depressed/I am moderately anxious or depressed/I am severely anxious or depressed/I am extremely anxious or depressed
EQ5D5L: VAS	We would like to know how good or bad your health is TODAY. This scale is numbered from 0 to 100. 100 means the best health you can imagine. 0 means the worst health you can imagine. (0–100)
PROMIS anxiety Tscore	t-score for anxiety derived from the PROMIS Depression and Anxiety Short Form 4a
PROMIS depression Tscore	t-score for depression derived from the PROMIS Depression and Anxiety Short Form 4a
EQ5D5L utility index DE	This index was computed based on the five single EQ5D5L items using the German value data set.
Oxford Shoulder Score	The Oxford Shoulder score is based on 12 items evaluated on a 5-point Likert scale. (0–48)
Perceived change (PC) Shoulder Pain	The three items were recorded as specific answers to the three aspects ‘Shoulder Pain’, ‘Shoulder Function’ and ‘Quality of Life’ in response to the single question ‘How are you today compared to before surgery?’ allowing the response categories ‘much worse’, ‘slightly worse’, ‘unchanged’, ‘somewhat better’ and ‘much better’.
PC Shoulder Function	
PC Quality of life	
Return to work	When did you return fully to work after the operation?

Note: The last four questions were only included at follow up.

PROM, patient-reported outcome measure; PROMIS, Patient-Reported Outcomes Measurement Information System; VAS, Visual Analogue Scale.

Patients were invited to clinical assessments at baseline and after 6 and 12 months. The baseline assessment was performed prior to surgery. The CROMs collected and included in this paper are shown in table 3. The pain and daily activity components of the Constant score^24^ and the pain level were based on patient interview. The strength and motion components were based on quantitative assessments. The Shoulder Stiffness Scale (SSS) is a monitoring tool for postoperative shoulder stiffness which has recently been investigated as a prognostic factor for return to sport.^25^ It combines patient interview and assessments. The Apley test is based on a clinical examination. Lost to follow-up rates were 5% and 9%.

**Table 3:** CROMs measured during follow up and at baseline

CROM	Question/response options/measurement
Shoulder Stiffness Scale (SSS)	The Shoulder Stiffness Scale is calculated from three items on pain (sss1: 0–3), on subjective range of movement limitation in daily activities (sss2: 0–3), and on side-to-side difference in passive external rotation in adduction and glenohumeral abduction (sss3: 0–4) resulting in a score between 0 and 10.
CS pain	NRS from 0 to 10 with 10 explained as ‘maximum pain’. Transformed to a range from 0 to 15.
CS daily activity	1. Is your occupation or daily living limited by your shoulder? No=4, Moderate limitation=2, Severe limitation=0; 2. Are your leisure and recreational activities limited by your shoulder? No=4, Moderate limitation=2, Severe limitation=0; 3. Is your night sleep disturbed by your shoulder? No=2, Sometimes=1, Yes=0; 4. State to what level you can use your arm for painless, reasonable activities. Waist=2, Xiphoid (sternum)=4, Neck=6, Head=8, Above head=10
CS motion	Range of movement. 1. Forward flexion and 2. Abduction: 0–30 0 pts; 31–60 2 pts; 61–90 4 pts; 91–120 6 pts; 121–150 8 pts; >150 10 pts. 3. External Rotation: Hand behind head and elbow forward 2 pts; Hand behind head and elbow back 4 pts; Hand above head and elbow forward 6 pts; Hand above head and elbow back 8 pts; Full elevation of arm 10 pts. 4. Internal Rotation (Dorsum hand to): Thigh 0 pts; Buttock 2 pts; Sacroiliac joint 4 pts; Waist 6 pts; T12 8 pts; Between shoulder blades 10 pts.
CS strength	Average strength over five pulls.
Constant score	Sum of the previous four items.
Pain level NRS	Patient’s highest pain level experienced in the affected shoulder during ordinary activities within the last 24 hours. (0–10)
Apley test IR up to L3	Apley Scratch Test (passed/failed)

CROM, clinician-reported outcome measures; CS, Constant score; IR, internal rotation; NRS, Numerical Rating Scale; pts, point.

A few additional outcomes are shown in box 1. They are based on routine clinical examinations. However, adverse events (AEs) could additionally be reported by the patient at the 12-month and 24-month questionnaire.^26^ All these additional outcomes are considered as CROMs in this paper. The scale of some outcomes was reversed to ensure that high values represent always a favourable outcome. Baseline characteristics used in this paper are listed in online supplemental table 1a. All baseline characteristics were measured on binary, ordinal or continuous scales.

Box 1 **Additional outcomes**

- Stiffness AE within 6 months.
- AE recurrent defect within 12 months.
- Stiffness within 6 months (AE&ROM).
- Full rec. defect within 24 months.

AE, adverse events; ROM, range of movement.

### Analytical strategy

#### Definition of potential indicators

All single outcome measurements during the follow-up of the patients were considered as potential indicators. Within the methodological framework used in this paper, there is no need to distinguish between using the raw values or the change from baseline—provided that any case-mix adjustment includes the baseline value of the outcome (see also online supplemental file 1). Overall, there were 49 potential indicators based on PROMs and 24 based on CROMs.

#### Selection of variables for case-mix-adjustment

All baseline characteristics in table 1a and all baseline measurements of PROMs and CROMs were considered as overall 54 potential variables for case-mix adjustments. Five different approaches for selection of variables were considered. The primary approach was based on an indicator-specific preselection of variables with predictive value for the indicator. This reflects a practice often used in case-mix adjustment. The lasso method^27^ was used for this automatic and indicator-specific preselection. Two choices of fixed subsets were considered as alternative approaches, focusing on variables identified as prognostic in previous research.^28 29^ The first choice included characteristics typically available in the clinical routine documentation: age, sex, American Society of Anesthesiologists (ASA) score, the four tear severity indicators and duration of shoulder complaints. The second choice focused on patient outcomes reported at baseline and included the Pain Numerical Rating Scale score, the EQ5D5L Visual Analogue Scale (VAS) and the two PROMIS scores on anxiety and depression in addition to age, sex and ASA score. For both choices the baseline value of the indicator was added if the indicator was included in the baseline assessment. As extreme approaches the adjustment for all baseline characteristics and no adjustment were considered.

#### Comparison of discriminative power of potential indicators

The discriminative power of a potential indicator reflects the ability of the indicator to distinguish between the clinics. It was quantified by the portion of the population variation of the indicator which can be explained by the clinics (after adjustment for case-mix). The 15 indicators with maximal power were determined, separately for those based on PROMs or on CROMs, respectively.

In order to give a visual impression about the discriminative power, for some selected indicators the centre-specific distributions were depicted graphically in more detail.

#### Impact of case-mix-adjustment on clinic variation

Clinic variation was described by the SD of the clinic-specific standardised mean values. The percentage reduction in clinic variation due to case-mix adjustment (relative to no case-mix adjustment) was reported for the most promising indicators identified in the previous step and the four approaches to variable selection for case-mix adjustment.

#### Impact of case-mix-adjustment on position of the clinics

In general, the standardised mean value of a clinic determines the (vertical) position of a clinic in a funnel plot. To evaluate the impact of the choice between two approaches to selection of variables for case-mix adjustment, the average and maximal absolute difference in the standardised mean values between the two approaches across all clinics was determined. The values were then expressed as a percentage of the range of the standardised mean values of the first approach, reflecting the relative change observable in a funnel plot. Selection of all variables was not included in these comparisons.

#### Examples of clinic comparisons

Funnel plots were presented for four indicators identified as promising in the previous steps and for all approaches to selection of variables for case-mix-adjustment except for selecting all variables.

#### Sensitivity analysis

A sensitivity analysis assessed whether considering single surgeons instead of clinics as care providers impacted the results.

#### Reporting

Reporting followed the recommendation of the Strengthening the Reporting of Observational Studies in Epidemiology (STROBE) statement.^30^

#### Statistical methods

The statistical methods used are described in online supplemental file 1.

## Results

### Distributions at baseline and at follow-up time points

Online supplemental table 1a-d depicts the distribution of all potential variables for case-mix-adjustment and of all outcomes measured only once over time.

Figure 1 depicts the distribution of all outcomes measured at several time points. All outcomes improved over time. For several outcomes the vast majority of patients was close to the most favourable value already after 12 months and in particular after 24 months. This implied limited variation across patients and clinics.

**Figure 1:**
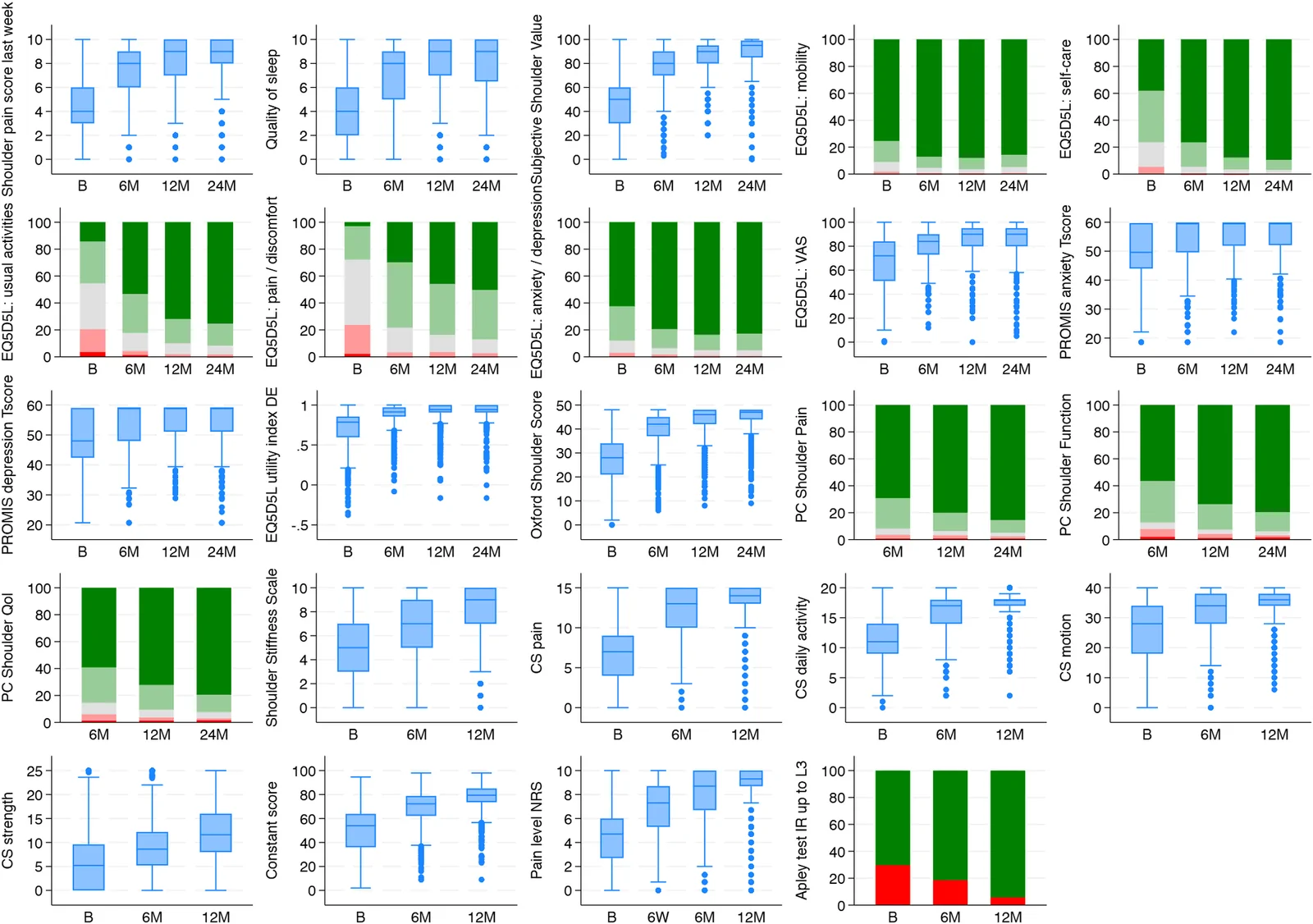
Distribution of all outcomes measured at several time points. For all outcomes, higher values refer to a more favourable state. In the stacked bar charts, the possible values are sorted from least favourable (bottom) to most favourable (top). B, baseline; CS, Constant score; IR, internal rotation; NRS, Numerical Rating Scale; PC, perceived change; PROMIS, Patient-Reported Outcomes Measurement Information System; VAS, Visual Analogue Scale.

### Comparison of the discriminative power of all potential indicators

In a preliminary comparison of the approaches to variable selection for case-mix adjustment, the lasso-based preselection resulted typically in power values close to the lowest values observed. Therefore, with respect to describing uncertainty by CIs and ranking of indicators, the focus was set on this specific approach. Figure 2 presents the 15 indicators based on PROMs or CROMs, respectively, with the highest discriminative power.

**Figure 2:**
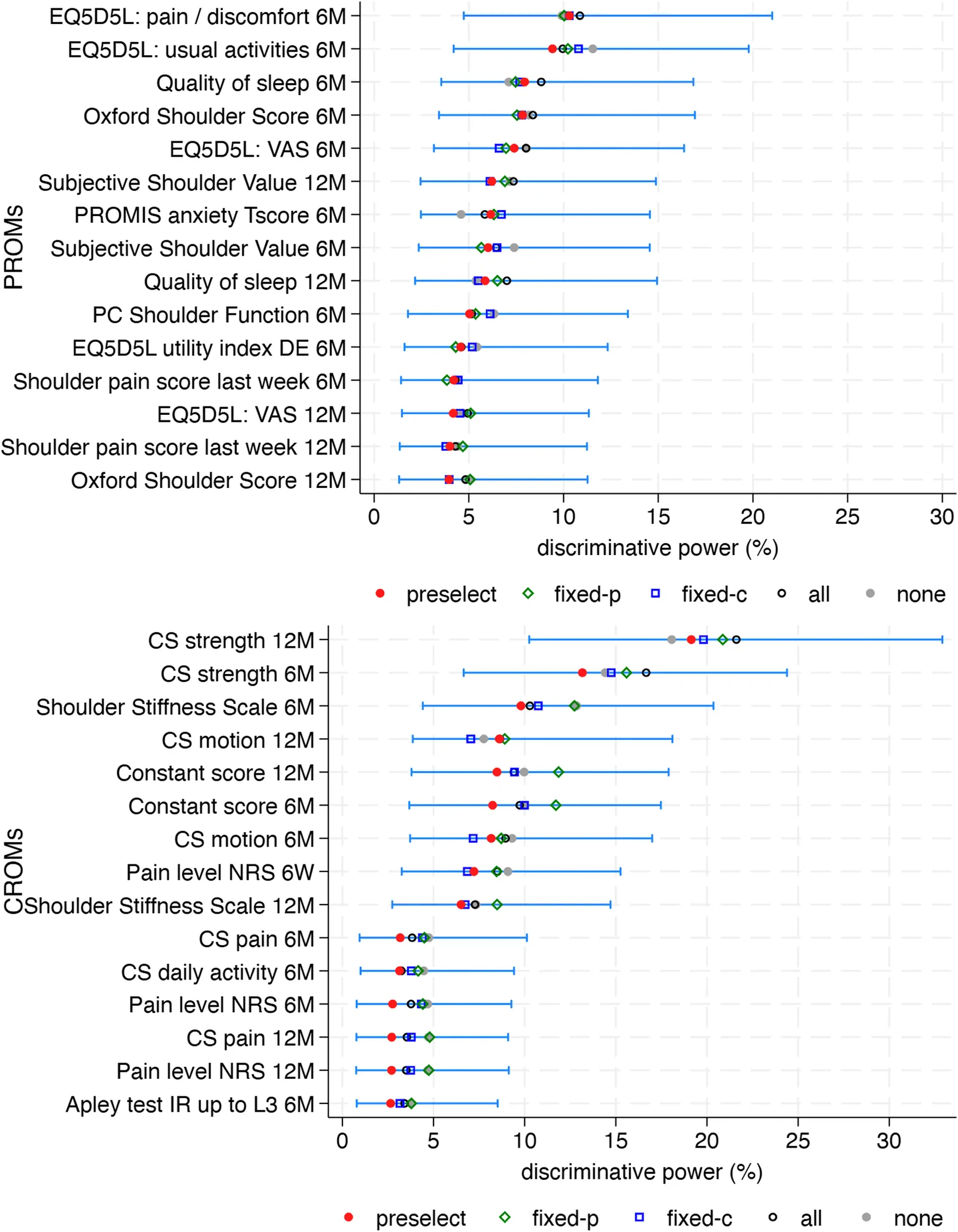
The 15 PROMs and CROMs, respectively, with maximal discriminative power when using automated preselection. The points refer to the different adjustment approaches. The CIs refer to automated preselection. All, all baseline variables; CROMs, clinician-reported outcome measures; CS, Constant score; fixed-c, fixed selection of clinical variables; fixed-p, fixed selection of PROMs; IR, internal rotation; none, no adjustment; NRS, Numerical Rating Scale; PC, perceived change; preselect, automated preselection; PROMs, patient-reported outcome measure; PROMIS, Patient-Reported Outcomes Measurement Information System; VAS, Visual Analogue Scale.

Online supplemental figure 1 illustrates the discriminative power for four indicators with high power. For all four indicators, it can be observed that even if the population is split into low-risk and high-risk patients for the specific indicator, within each subpopulation distinct differences between the clinics can be observed such that the upper quartile in some clinics is lower than or close to the lower quartile in other clinics.

10 of the 15 PROMs shown in figure 2 are based on a measurement 6 months after surgery, five on a measurement 12 months after surgery, and none on measurements 24 months after surgery. For the CROMs shown in figure 2, six are based on measurements after 12 months, including the indicator with highest discriminative power. However, also the pain level after 6 weeks is among the 15 indicators with highest discriminative power.

### Impact of case-mix-adjustment on clinic variation

Figure 2 depicts already that the specific set of variables chosen for case mix adjustment has often little influence on the clinic variation, but that there are some indicators for which case-mix adjustment reduces the variation distinctly. Online supplemental figure 2 takes a closer look at the reduction in clinic variation due to case mix-adjustment. For most PROMs, the reduction is in the magnitude of 0% to 20%, that is, of rather moderate magnitude. In addition, a few variables can be sufficient to come close to the maximally possible reduction. For some CROMs, the reduction can be somewhat larger, and a few variables need not to be sufficient. Potential explanations for the moderate magnitude are investigated in online supplemental file 2.

### Impact of case-mix-adjustment on position of the clinics

The use of any of the four case-mix adjustment approaches implies for most indicators an average change in the relative position in a funnel plot in a magnitude of 5% to 10% compared with no adjustment (online supplemental figure 3). Focusing on the maximal change across all clinics, the change rarely exceeds 30%.

### Examples of clinic comparisons

The results of the previous sections suggest a wide variety of potential indicators with high discriminative power. Four choices are considered as examples in figure 3: a generic PROM (‘usual activities’ item of the EQ5D5L), a shoulder specific PROM (Oxford shoulder score), a clinical assessment (Constant score strength) and an outcome based on patient interview and assessments during a clinical examination (SSS). All four indicators are based on the measurements after 6 months.

**Figure 3:**
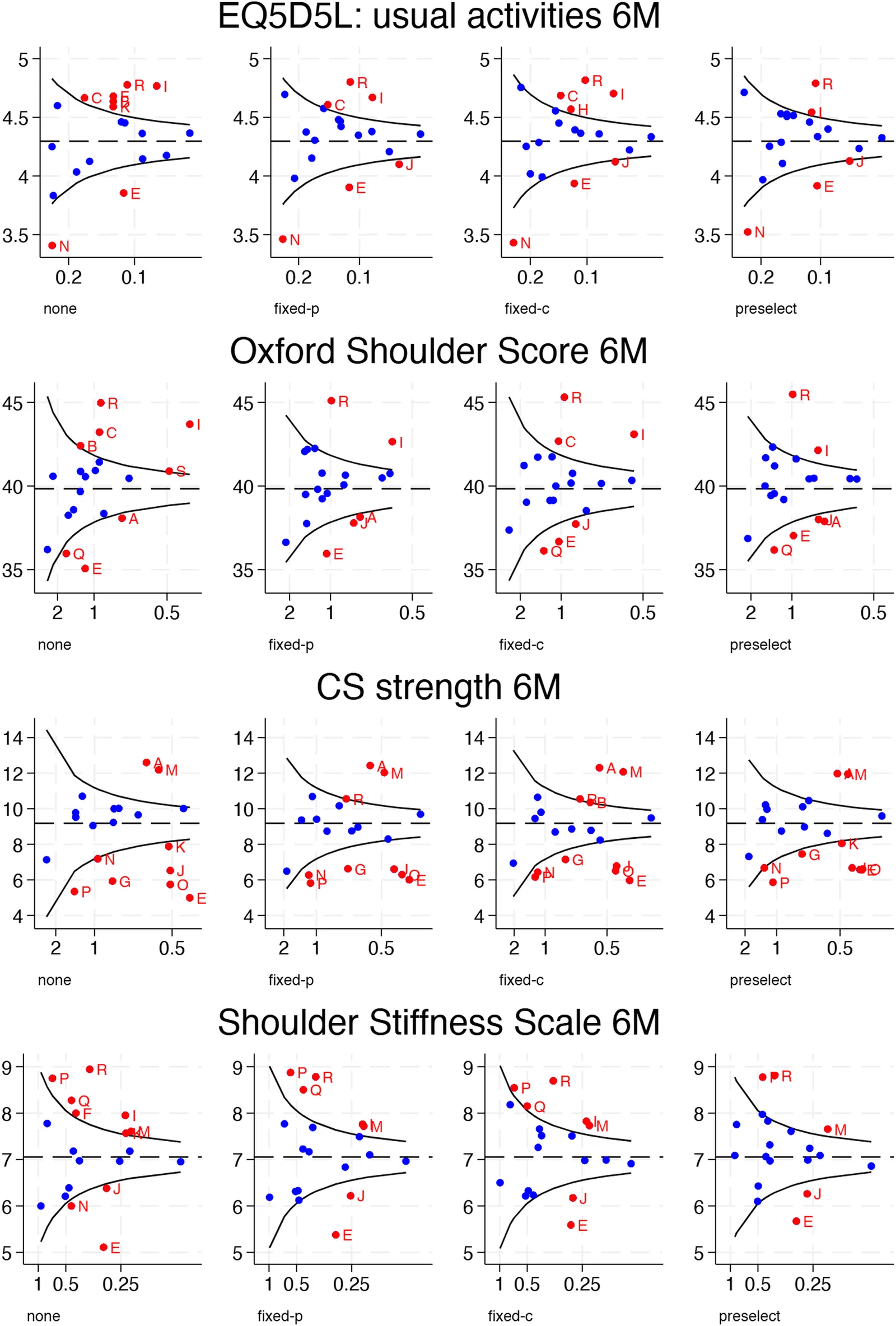
Funnel plots for four potential indicators and four approaches to variable selection for case-mix adjustment. The dots refer to the standardised mean values of the single centres. The y-axes refer to the scale of the indicator. The x-axes refer to the SE of the standardised mean values and use a reciprocal scale. CS, Constant score; fixed-c, fixed selection of clinical variables; fixed-p, fixed selection of PROMs; none, no adjustment; preselect, automated preselection.

Between five and nine clinics are identified as falling outside of the normal range. Adjustment can reduce the number of clinics identified, but the type of adjustment has a limited effect. The two PROMs identify roughly the same clinics, and the most prominent outliers (R, I, E and N) play a similar role when using SSS. Using the clinical assessment of strength, there is only a limited overlap in the clinics identified with the other three analyses. Clinic P moves even from a high-performing clinic according to SSS to a low-performing clinic according to Constant score strength.

### Sensitivity analyses

The comparison of surgeons instead of clinics had little impact on the general results with respect to discriminative power (online supplemental file 3). However, the need for case-mix adjustment increased.

## Discussion

### Summary of results

Our investigation reveals that some of the PROMs as well as CROMs considered allow us to demonstrate rather distinct differences in the outcome across providers of ARCR, which do not seem to be explainable only by differences in the patient populations. Hence, they allow us to identify differences in care delivery. In addition, the required case-mix adjustment may be achieved using only a small set of variables that are already routinely collected or can be obtained with limited additional effort, such as baseline PROM values.

It might be somewhat surprising that two items of the generic instrument EQ5D5L seem to be more powerful to discriminate between providers than the joint-specific instrument OSS. This may be due to ceiling effects for the OSS observable already 6 months after surgery (figure 1) which are present only to a lower degree for the two items of the EQ5D5L. Ceiling effects in the OSS starting already 6 months after surgery have also been identified in a recent review of the measurement properties of the OSS^31^ based on the results of a single study.^32^

### Practical impact for comparing providers of ARCR

There is a wide range of PROMs and CROMs, all of which can reveal substantial differences in outcome across providers when collected during follow-up. This offers a lot of opportunities to design quality management initiatives in shoulder surgery. Our investigation can be informative for three aspects: Choice of follow-up time points, choice of outcomes and choice of variables for case-mix adjustments.

#### Choice of follow-up time points

As regards the follow-up duration, our analyses indicate that outcome measurements at 6 months provide discriminative power comparable to those at 12 months, whereas measurements at 24 months show distinctly lower discriminative ability. In this context, a 6-month follow-up appears preferable for routine benchmarking: it captures between-provider differences while minimising loss to follow-up—which was higher at 12 months in our cohort. Moreover, shorter follow-up enables faster feedback to providers. Notably, even pain assessed as early as 6 weeks after surgery already discriminates between providers and may serve as an early signal of outcome differences. Nevertheless, we cannot exclude that additional patient-relevant differences might emerge only with longer-term follow-up (eg, 5–10 years), which would require dedicated longitudinal studies.

#### Choice of outcomes

As regards the choice of outcomes, there is a temptation to use the ranking in discriminative power observed in our investigation. However, this idea is of limited value, as the CIs are wide and there are still many options for the best indicator. More essential for the choice of indicators may be the question whether the conceptual differences between outcome measures imply also a difference in the ranking of the providers. This question was not investigated systematically, but the examples of clinic comparisons provided suggest that there may be a substantial difference between ‘quality’ assessed by clinical examination and ‘quality’ assessed by patient reporting.

In general, PROMs may be preferred to CROMs, as the latter may be influenced by clinicians—they may differ with respect to performing the same assessment. However, the ‘best’ CROM identified in our investigation—Constant score strength—can be based on a rather objective assessment procedure and may hence be less problematic. Our results suggest that Constant score strength may be more effective than PROMs in discriminating between providers.

The appearance of two items of the EQ5D5L among the outcomes with highest discriminative power may support the use of the EQ5D5L as part of the current SOMDS, aiming at a tool applicable across many orthopaedic subdisciplines.^4 33^

#### Choice of variables for case-mix adjustments

Our results suggest that case-mix adjustment is necessary for most of the potential outcome indicators considered, but that the impact on the ranking is rather moderate. The latter makes it difficult to judge the quality of different approaches to the selection of variables for case-mix adjustment. The uniformity of the results on the impact on the position of clinics when comparing different sets in online supplemental figure 3 indicates that we mainly observe stochastic uncertainty and we do not catch systematic differences. Hence it can only be concluded that some case-mix adjustment is necessary for most indicators and that for many indicators there is no evidence against using some standard variables from clinical routine.

### Limitations

Due to considering a large number of potential indicators it was necessary to apply an automatic selection of variables for case-mix adjustment. This precluded the careful analysis of the existence of non-linear or non-additive effects as well as the use of background knowledge. This may imply an overestimation of the discriminative power. Similarly, the specific ordinal or binary scale of some outcomes as well as ceiling effects were ignored, which may imply a suboptimal judgement of their value.

The question of the impact of the choice of different approaches to variable selection for case-mix adjustment could only be addressed using empirical examples. It remains unclear, whether the differences observed are due to random fluctuations or represent systematic differences. This question can only be addressed in larger samples. The same holds for the question about the impact of using different potential indicators.

The illustrative examples of funnel plots focused on indicators with high observed discriminative power. This data-driven selection may introduce overoptimism with respect to their true value.

The comparison of providers was based exclusively on mean values. Other distributional characteristics, for example, percentiles, may be more informative, allowing to catch occasional low-level performance instead of average performance. Corresponding systematic comparisons are part of further ongoing research.

Participation of clinics in the ARCR_Pred study was voluntary. The amount of provider variation may differ when considering all clinics offering ARCR in Switzerland.

### Comparison with other surgical disciplines

Previous studies have already shown that it is possible to detect differences in outcomes across providers of surgical care using PROMs. Differences across providers of hip replacement could be demonstrated using the EQ5D5L^34^—a generic instrument—as well as when using the Hip disability and osteoarthritis outcome score^35^—a hip-specific instrument.^10^ Similarly, the Knee Injury and Osteoarthritis Outcome Score^36^ allowed to demonstrate differences across providers of knee replacement.^9^ The Health and Activities Limitations Index^37^ allowed to demonstrate provider differences in bariatric surgery^7^ whereas an attempt to demonstrate provider variation after lumbar fusion using the Oswestry Disability Index failed.^8^ Unfortunately, the reporting of the provider variation in these studies is too heterogeneous to assess whether provider variation is specifically high or low in shoulder surgeons.

### Outlook

The design of quality monitoring programmes in shoulder surgery requires many crucial decisions which cannot be made solely based on empirical investigations. It is important to take into account the preferences, values and interests of various stakeholders. The empirical investigations in this paper check only one precondition of potential outcome quality indicators: A detectable variation across providers.

It would be beneficial to inform such discussions by clarifying whether outcomes conceptually different and/or measured at different time points may nevertheless reflect the same dimension of outcome quality. If such quality dimensions can be identified, it might be possible to develop summary indicators for specific dimensions.

Our investigation may also point to the need to investigate further shoulder or RC-specific PROMs which suffer less from ceiling effects and hence may allow to detect outcome differences at later follow-up time points. These may be chosen from existing instruments^38 39^ but there may be a need to develop specific instruments focusing on more subtle aspects of long term health impact. In addition, comparisons with other surgical fields would be informative to judge the generalisability of our findings.

## Conclusions

It is possible to demonstrate differences in the outcome of providers offering ARCR using PROMs or CROMs, which do not seem to be explainable only by differences in the patient populations. Hence, they offer the opportunity to inform provider comparisons. There is a need for case-mix adjustment, but as the impact is limited, it often seems to be justified to use few variables routinely available.

## Supplementary material

10.1136/bmjoq-2026-004516 online supplemental file 1

10.1136/bmjoq-2026-004516 online supplemental file 2

10.1136/bmjoq-2026-004516 online supplemental file 3

10.1136/bmjoq-2026-004516 online supplemental file 4

10.1136/bmjoq-2026-004516 online supplemental file 5

## Data Availability

Data may be obtained from a third party and are not publicly available.
