# Supplementary material for: Identifying patient-reported and clinician-reported outcome measures to compare providers of arthroscopic rotator cuff repair: insights obtained from a Swiss multicentre cohort study
Source: BMJ Open Qual. 2026 Nov 11;15(4):e004516. doi: 10.1136/bmjoq-2026-004516 (PMC13756451; doi:10.1136/bmjoq-2026-004516)
Supplement: Supplementary file 1 [file bmjoq-15-4-s001.pdf]

Supplemental File 1: Statistical methods

Regression modelling

The analyses made use of a linear regression model with the indicator of interest as outcome and all baseline variables selected for case-mix adjustment as covariates. All outcomes and all covariates were regarded as continuous variables. Variables originally measured on an ordinal scale entered the model with an equidistant numerical coding. Depending on the type of analysis, a clinic indicator may have entered the model as random or fixed effect. In case of adding a random effect, estimation was based on the restricted maximum likelihood (REML) method.

Estimation of the discriminative power of an indicator

To determine the portion of the population variation of an indicator which can be explained by the clinics (after adjustment for case-mix), the clinic indicator was added to the model as a random effect. The estimated variance of this random effect was expressed as percentage of the sum of this variance and the variance of the residuals. This sum reflected the variance of the indicator which cannot be explained by the covariates. Confidence intervals were based on the Wald technique applied to the logit transformed percentages.

Estimating the standard deviation of the case-mix adjusted clinic-specific mean values

To estimate the standard deviation of the case-mix adjusted clinic-specific mean values, the variable clinic was added to the model as random effect and the standard deviation of this random effect was estimated.

Direct standardization

Direct standardization was considered to implement case-mix adjustment. This approach determines for each clinic the mean value of the indicator to be expected if the clinic would have managed all patients of the study – called the standardized mean value.

Direct standardization was based on adding the variable clinic as a fixed effect to the regression model. The standardized mean value of a clinic was obtained by computing for each patient the predicted value from the model assuming that all patients share the intercept of the clinic and by taking the average over all predicted values. A formal description of this approach is outlined in Appendix 1.

Funnel plots

Funnel plots [1] were based on plotting the observed standardized mean values against the standard error of these estimates. The standard error was determined by drawing 250 bootstrap samples with clinic as stratification factor [2]. A reciprocal transformation was applied to the horizontal axis. The expected range was defined as +/- 2 times the standard error added to the overall population mean.

Handling of missing values

As the follow up of the patients was rather complete and item-nonresponse was rare, no attempts were made to fill the data by imputation. All analyses refer to an available case analysis, i.e. all patients participating at a specific timepoint are included in the analysis.

Baseline assessments were complete except of three patients with lack of the strength measurement of the Constant score. In these three patients, the value predicted based on a regression on the three other components of the Constant score was imputed. The baseline variable “current occupational position” was not defined in patients currently not occupied. Here a “missing indicator” approach [3] was used. This means that the variable was set to 0, and the variable “currently occupied?” served as missing indicator. This way it was possible to include all subjects always in the regression analysis.

Appendix 1: Formal description of direct standardization

Let Y_{ci} denote the indicator of interest for patient i at clinic c, and let X_{ci} denote the vector of adjustment variables for patient i at clinic c. Direct standardization requires to fit the regression model

$$Y_{ci} = \beta_c + \beta\, X_{ci} + \epsilon_{ci}$$

The standardized mean value of clinic c can then be computed as

$$\bar{Y}_c^S \quad = \quad \beta_c + \frac{1}{N}\sum_{c\prime=1}^{N_C}\sum_{i=1}^{n_{c\prime}} \beta X_{c\prime i}$$

with $n_{c\prime}$ denoting the number of patients at clinic c', N_C the number of clinics, and N the overall number of patients.

Appendix 2: Equivalence of direct standardization of raw values and change scores

Let Y_{ci} be a follow up measurement and B_{ci} the corresponding baseline value. Direct standardization of Y requires to fit the regression model

$$Y_{ci} = \beta_c + \gamma B_{ci} + \beta' X_{ci} + \epsilon_{ci}$$

whereas direct standardization of the change scores $C_{ci} = Y_{ci} - B_{ci}$ requires to fit the regression model

$$C_{ci} = Y_{ci} - B_{ci} = \tilde{\beta}_c + \tilde{\gamma} B_{ci} + \tilde{\beta}' X_{ci} + \tilde{\epsilon}_{ci}$$

This implies that the regression coefficients are identical except of that $\tilde{\gamma} = \gamma - 1$.

This implies that

$$\bar{Y}_c^S = \beta_c + \gamma \bar{B} + \frac{1}{N}\sum_{c\prime=1}^{N_C}\sum_{i=1}^{n_{c\prime}} \beta' X_{c\prime i}$$

and

$$\bar{C}_c^S = \tilde{\beta}_c + \tilde{\gamma} \bar{B} + \frac{1}{N}\sum_{c'=1}^{N_C}\sum_{i=1}^{n_{c'}} \tilde{\beta}' X_{c' i} = \bar{Y}_c^S - \bar{B}$$

i.e. centre-specific values $\bar{Y}_c^S$ *and* $\bar{C}_c^S$ differ only by the constant $\bar{B}$. Consequently, the positioning of the centres in a funnel plot will be identical.

1. Spiegelhalter DJ. Funnel plots for comparing institutional performance. Statistics in Medicine. 2005 ;24(8):1185-1202.

2. Neuburger J, Cromwell DA, Hutchings A, Black N, van der Meulen JH. Funnel plots for comparing provider performance based on patient-reported outcome measures. BMJ Qual Saf. 2011 December;20(12):1020-1026.

3. Miettinen OS. *Theoretical Epidemiology: Principles of Occurrence Research in Medicine*. Wiley; 1985.
