# Supplementary material for: Identifying patient-reported and clinician-reported outcome measures to compare providers of arthroscopic rotator cuff repair: insights obtained from a Swiss multicentre cohort study
Source: BMJ Open Qual. 2026 Nov 11;15(4):e004516. doi: 10.1136/bmjoq-2026-004516 (PMC13756451; doi:10.1136/bmjoq-2026-004516)
Supplement: Supplementary file 2 [file bmjoq-15-4-s002.pdf]

Supplemental File 2: Possible explanations for a moderate need for risk adjustment

A first explanation for a moderate need for risk adjustment can be that the patient populations are very similar across the clinics. Figures S1.1 suggests that this is not the case. There are several baseline characteristics which allow to discriminate between the clinics to a substantial degree. The clinics differ mainly with respect to clinical characteristics such as previous interventions, shoulder stiffness or tear severity as well as with respect to patient reported characteristics such as duration of shoulder complaints, alcoholic beverages, smoking and quality of life.

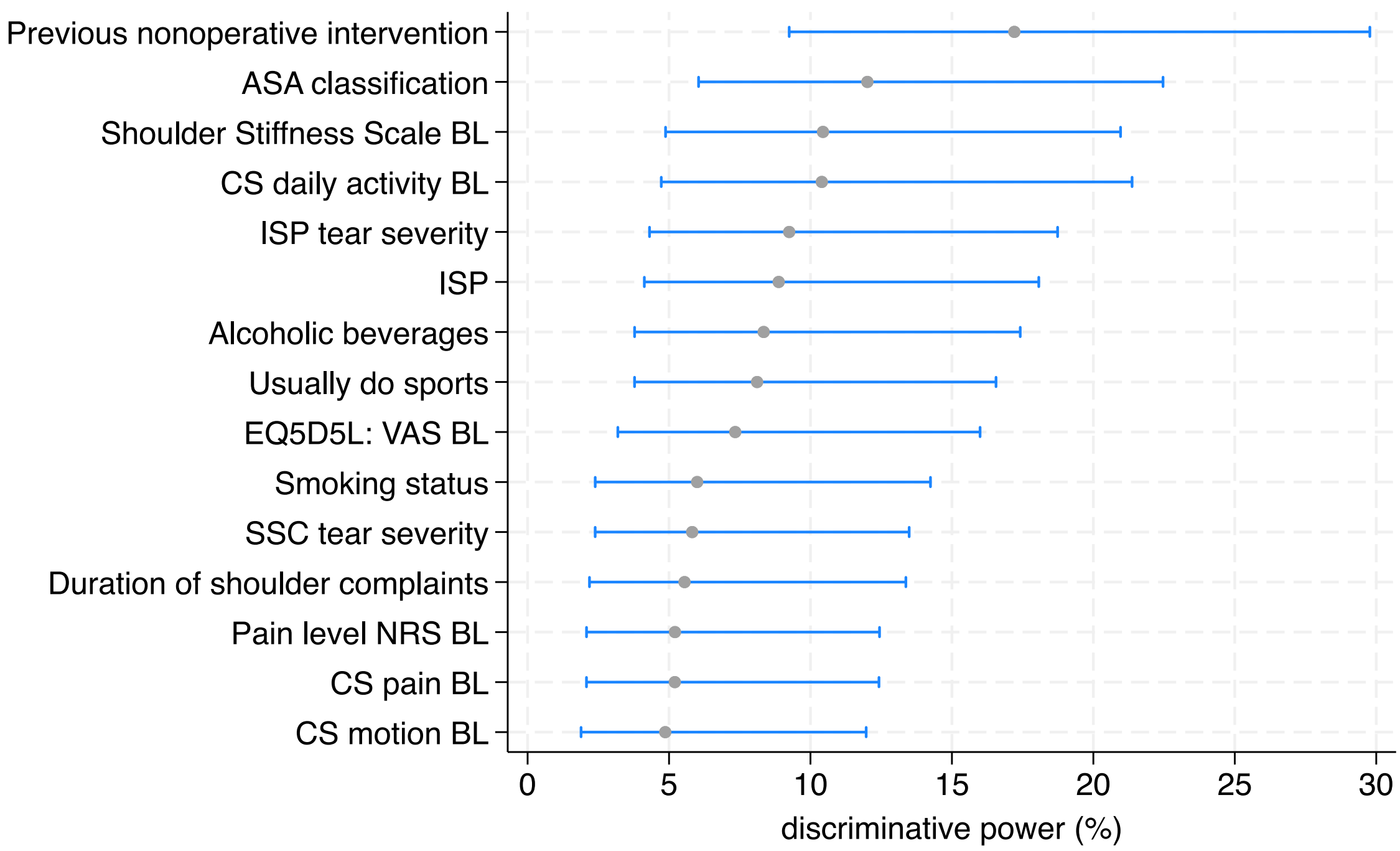

Figure S1.1: The 15 baseline variables with maximal discriminative power. No adjustment for other variables is performed. CS; Constant score. ASA: American Society of Anesthesiologists; BL: Baseline; ISP: M. infraspinatus; VAS: Visual analog scale; NRS: Numerical rating scale; SSC: M. subscapularis

A second explanation can be a lack of association between baseline characteristics and the indicators of interests. However, this does not seem to be the case. Among the 30 indictors identified in Figure 2 of the main manuscript 13 can be predicted based on the preselected variables with an adjusted R^2 value above 0.2 and further 13 with a value above 0.15.

However, risk adjustment can only have an effect if there are variables with distributions differing between clinics which are also prognostic for the indicator of interest. Figure S1.2 indicates that there may be a lack of such variables. For both indicators considered there are only few baseline variables which have a high discriminative power, and which also correlate with the indicator.

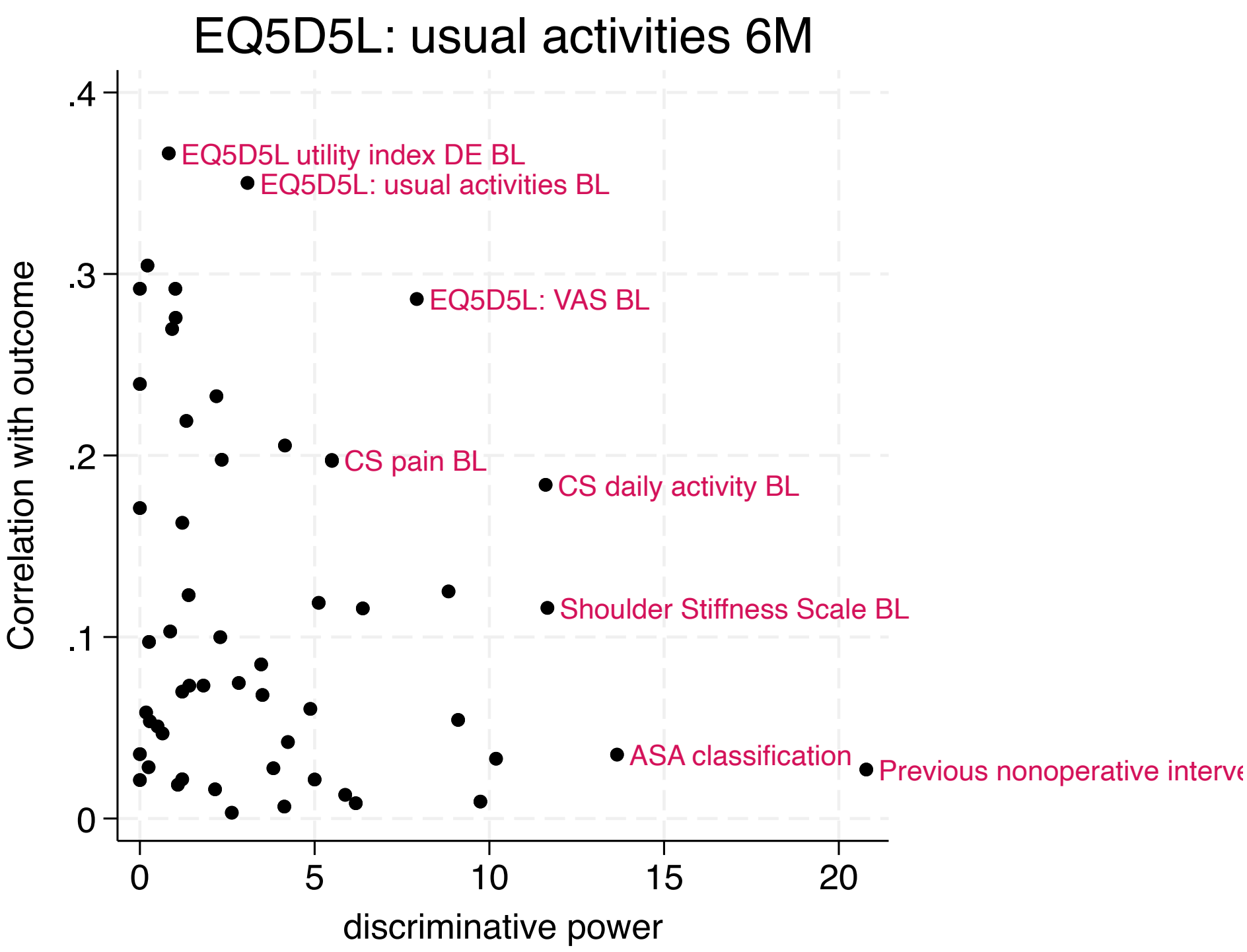

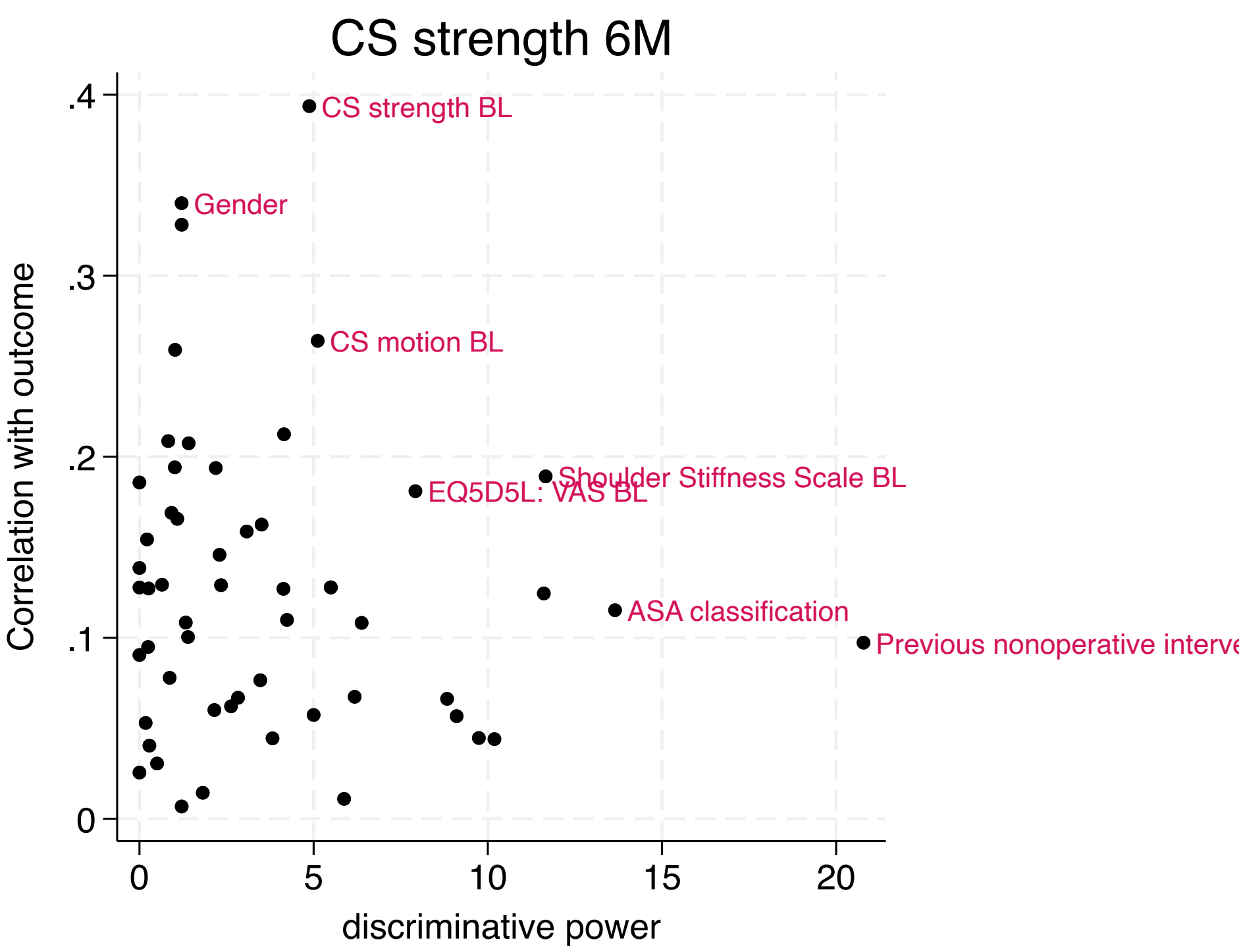

Figure S1.2: Scatterplots of the absolute value of the correlation with the outcome (for two selected outcomes) vs the discriminative power for all baseline variables. Each dot reflects one baseline variable. Some baseline variables with high correlation, with high discriminative power or both moderate correlation and power are labelled. CS: constant score; 6M; 6 months. BL: baseline; ASA: American Society of Anesthesiologists
