# Supplementary material for: Identifying patient-reported and clinician-reported outcome measures to compare providers of arthroscopic rotator cuff repair: insights obtained from a Swiss multicentre cohort study
Source: BMJ Open Qual. 2026 Nov 11;15(4):e004516. doi: 10.1136/bmjoq-2026-004516 (PMC13756451; doi:10.1136/bmjoq-2026-004516)
Supplement: Supplementary file 3 [file bmjoq-15-4-s003.pdf]

Supplemental File 3: The impact of considering surgeons instead of clinics as providers

Figure S2.1 illustrates that many indicators show a substantial discriminative power also if surgeons are considered as providers. No substantial shift in the indicators with maximal discriminative power can be observed. Figure S2.2 indicates that there is an increased need for risk adjustment, but that – in particular for PROMs – few variables can be sufficient.

As visible from Table 1 in the main manuscript, in seven out of the 19 clinics all care was provided by one surgeon. This may partially explain the similarity of the results of this sensitivity analysis compared to the analysis presented in the main part of the manuscript.

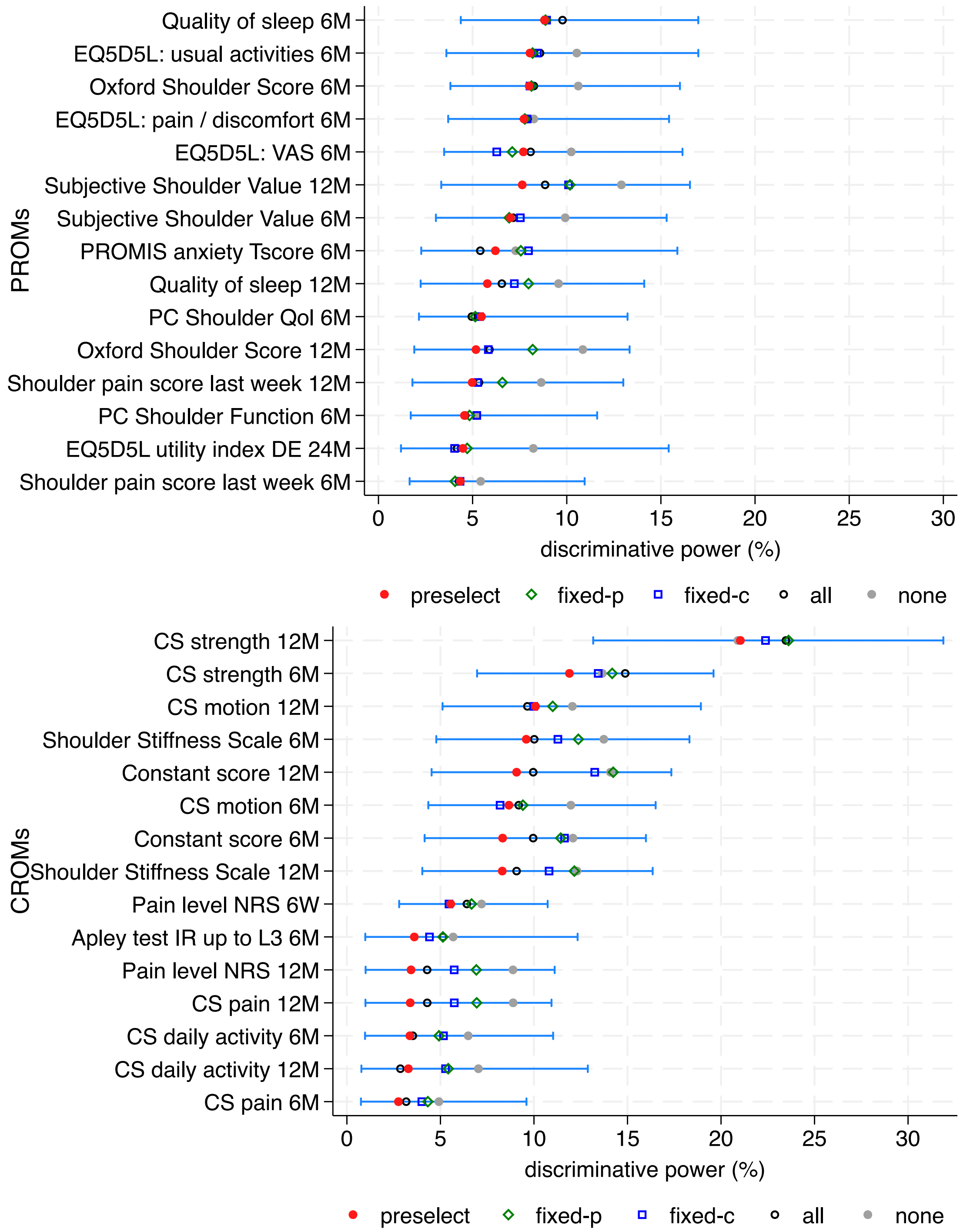

Figure S2.1: The 15 PROMs and CROMs, respectively, with maximal discriminative power when surgeons are considered as providers and when using automated preselection. The points refer to the different adjustment approaches. The confidence intervals refer to automated preselection. CS: Constant score; PROMIS: Patient reported outcomes measurement information system; 6M: 6 months; 12M: 12 months; 6W: 6 weeks; IR: internal rotation; NRS: Numerical rating scale; fixed-p: fixed selection of PROMs; fixed-c: fixed selection of clinical variables; none: no adjustment; preselect: automated preselection; all: all baseline variable;

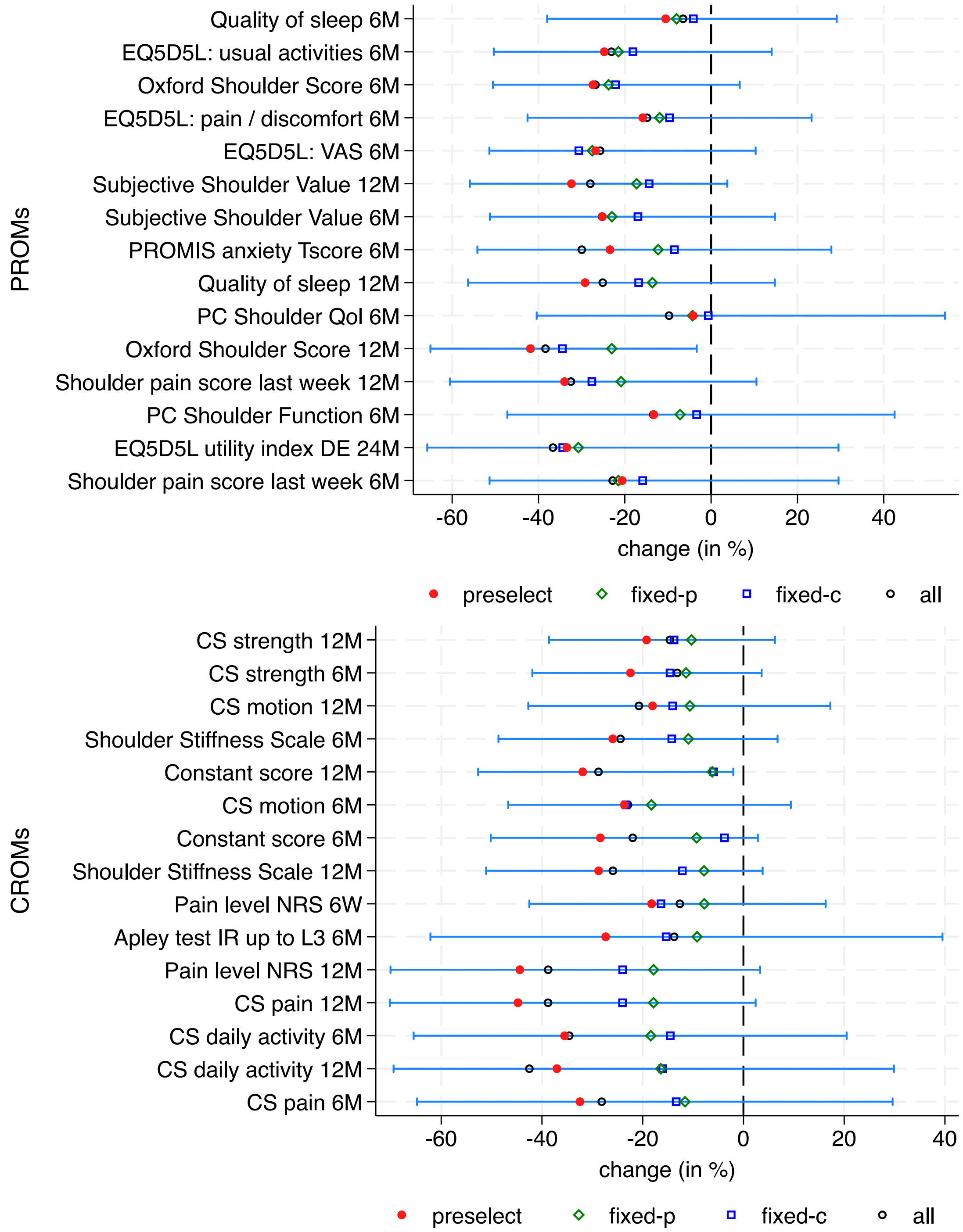

Figure S2.2: The reduction in the standard deviation of the surgeon-specific mean values due to case-mix adjustment compared to no adjustment for the indicators identified in Figure S2.1. CS: Constant score; PROMIS: Patient reported outcomes measurement information system; 6M: 6 months; 12M: 12 months; 6W: 6 weeks; IR: internal rotation; NRS: Numerical rating scale; fixed-p: fixed selection of PROMs; fixed-c: fixed selection of clinical variables; preselect: automated preselection; all: all baseline variables;.
