# Supplementary material for: Identifying patient-reported and clinician-reported outcome measures to compare providers of arthroscopic rotator cuff repair: insights obtained from a Swiss multicentre cohort study
Source: BMJ Open Qual. 2026 Nov 11;15(4):e004516. doi: 10.1136/bmjoq-2026-004516 (PMC13756451; doi:10.1136/bmjoq-2026-004516)
Supplement: Supplementary file 4 [file bmjoq-15-4-s004.pdf]

Supplemental Table 1a: Baseline characteristics and their distribution

Age at surgery (n=973)	
mean (sd)	57.3 (9.4)
5th-95th percentile	42.0-73.0
Gender (n=973)	
Female	362 (37.2%)
Male	611 (62.8%)
ASA classification (n=973)	
I	429 (44.1%)
II	472 (48.5%)
III	71 (7.3%)
IV	1 (0.1%)
Body Mass Index (BMI) (n=973)	
mean (sd)	26.8 (4.4)
5th-95th percentile	20.4-35.3
Overweight (n=973)	
No	349 (35.9%)
Yes	624 (64.1%)
Current smoker (n=973)	
No	769 (79.0%)
Yes	204 (21.0%)
Smoking status (n=973)	
Never smoked	575 (59.1%)
Smoked before	194 (19.9%)
Current smoker	204 (21.0%)
Alcoholic beverages (n=973)	
No	232 (23.8%)
Occasionally	645 (66.3%)
1x daily	82 (8.4%)
Several times a day	14 (1.4%)
One or more comorbidities (n=973)	
No	462 (47.5%)
Yes	511 (52.5%)
Number of comorbidities (n=973)	
mean (sd)	0.7 (1.0)
5th-95th percentile	0.0-3.0
Diabetes (n=973)	
No	930 (95.6%)
Yes	43 (4.4%)
Heart disease / High blood pressure (n=973)	
No	713 (73.3%)
Yes	260 (26.7%)
Usually do sports (n=973)	
No	221 (22.7%)

Yes	752 (77.3%)
Education level (n=973)	
Up to 9 years (Compulsory school)	129 (13.3%)
10-13 years (Apprenticeship / High school)	493 (50.7%)
13+ years (Higher / University, College)	351 (36.1%)
Currently occupied? (n=973)	
No	254 (26.1%)
Yes	719 (73.9%)
Current occupational position (n=719)	
Employee without leadership responsibilities	335 (46.6%)
In lower or middle management	165 (22.9%)
In upper or the highest management	80 (11.1%)
Self-employed or own small business	139 (19.3%)
Last occupational position (n=973)	
I have not been working yet	18 (1.8%)
Employee without leadership responsibilities	433 (44.5%)
In lower or middle management	221 (22.7%)
In upper or the highest management	122 (12.5%)
Self-employed or own small business	179 (18.4%)
Marital status (n=973)	
alone	283 (29.1%)
not alone	690 (70.9%)
Dominant side operated (n=973)	
Non-dominant	269 (27.6%)
Dominant	704 (72.4%)
Duration of shoulder complaints (n=973)	
mean (sd)	4.3 (1.5)
5th-95th percentile	2.0-6.0
Previous nonoperative intervention (n=973)	
No	349 (35.9%)
Yes	624 (64.1%)
Use of statin medication (n=973)	
No	842 (86.5%)
Yes	131 (13.5%)
Number of steroid infiltrations (n=973)	
mean (sd)	0.5 (1.0)
5th-95th percentile	0.0-3.0
Number of steroid infiltration (n=973)	
None	683 (70.2%)
1	171 (17.6%)
2	67 (6.9%)
3+	52 (5.3%)
Cause (judged by surgeon) (n=973)	
Purely degenerative	275 (28.3%)
More degenerative than traumatic	182 (18.7%)
More traumatic than degenerative	232 (23.8%)
Purely traumatic	284 (29.2%)

Trauma-related RC tear (n=973)	
Degenerative	457 (47.0%)
Traumatic	516 (53.0%)
SSC (n=973)	
No	513 (52.7%)
Yes	460 (47.3%)
SSP (n=973)	
No	72 (7.4%)
Yes	901 (92.6%)
ISP (n=973)	
No	573 (58.9%)
Yes	400 (41.1%)
SSC tear severity (n=973)	
Intact	513 (52.7%)
Partial	125 (12.8%)
Complete	335 (34.4%)
SSP tear severity (n=973)	
Intact	72 (7.4%)
Partial	177 (18.2%)
Complete	724 (74.4%)
ISP tear severity (n=973)	
Intact	573 (58.9%)
Partial	106 (10.9%)
Complete	294 (30.2%)
RC tear severity (intra-operative) (n=973)	
Partial tear	147 (15.1%)
Single full tear	255 (26.2%)
Two or three tendons (only one full)	143 (14.7%)
Massive tear (Gerber et al)	428 (44.0%)

ASA: American Society of Anesthesiologists; SSC: M. subscapularis; SSP: M. supraspinatus; ISP: M. infraspinatus; RC: Rotator cuff

Supplemental Table 1b: Distribution of PROMs at baseline

Shoulder pain score last week (n=973)	
mean (sd)	4.2 (2.2)
5th-95th percentile	1.0-8.0
Quality of sleep (n=973)	
mean (sd)	4.0 (2.5)
5th-95th percentile	0.0-9.0
Subjective Shoulder Value (n=973)	
mean (sd)	46.6 (19.3)
5th-95th percentile	10.0-80.0
EQ5D5L: mobility (n=973)	
1	2 (0.2%)
2	16 (1.6%)
3	70 (7.2%)

4	151 (15.5%)
5	734 (75.4%)
EQ5D5L: self-care (n=973)	
1	3 (0.3%)
2	49 (5.0%)
3	178 (18.3%)
4	373 (38.3%)
5	370 (38.0%)
EQ5D5L: usual activities (n=973)	
1	35 (3.6%)
2	165 (17.0%)
3	331 (34.0%)
4	303 (31.1%)
5	139 (14.3%)
EQ5D5L: pain / discomfort (n=973)	
1	22 (2.3%)
2	211 (21.7%)
3	471 (48.4%)
4	241 (24.8%)
5	28 (2.9%)
EQ5D5L: anxiety / depression (n=973)	
1	5 (0.5%)
2	24 (2.5%)
3	89 (9.1%)
4	248 (25.5%)
5	607 (62.4%)
EQ5D5L: VAS (n=973)	
mean (sd)	68.7 (19.8)
5th-95th percentile	30.0-95.0
Sick leave (n=711)	
full	226 (31.8%)
partial	39 (5.5%)
none	446 (62.7%)
PROMIS anxiety Tscore (n=973)	
mean (sd)	49.8 (8.9)
5th-95th percentile	34.2-59.7
PROMIS depression Tscore (n=973)	
mean (sd)	48.6 (8.8)
5th-95th percentile	34.2-59.0
EQ5D5L utility index DE (n=973)	
mean (sd)	0.7 (0.2)
5th-95th percentile	0.2-0.9
Oxford Shoulder Score (n=973)	
mean (sd)	27.3 (8.9)
5th-95th percentile	11.0-41.0

PROMIS : Patient reported outcome measurement information system. VAS: Visual analog scale

Supplemental Table 1c: Distribution of CROMs at baseline

Shoulder Stiffness Scale (n=973)	
mean (sd)	4.8 (2.5)
5th-95th percentile	1.0-9.0
CS pain (n=973)	
mean (sd)	6.8 (3.4)
5th-95th percentile	2.0-13.0
CS daily activity (n=973)	
mean (sd)	11.4 (3.8)
5th-95th percentile	5.0-17.0
CS motion (n=973)	
mean (sd)	26.1 (10.5)
5th-95th percentile	6.0-40.0
CS strength (n=970)	
mean (sd)	6.1 (5.9)
5th-95th percentile	0.0-17.6
Constant score (n=970)	
mean (sd)	50.4 (18.3)
5th-95th percentile	18.0-77.2
Pain level NRS (n=973)	
mean (sd)	4.5 (2.3)
5th-95th percentile	1.3-8.7
Apley test IR up to L3 (n=973)	
No	290 (29.8%)
Yes	683 (70.2%)

CS:Constant score; NRS: Numerical rating scale; IR: Internal rotation

Supplemental Table 1d: Distribution of outcomes measured once

Stiffness AE within 6 months (n=973)	
No	895 (92.0%)
Yes	78 (8.0%)
AE recurrent defect within 12 months (n=973)	
No	929 (95.5%)
Yes	44 (4.5%)
Stiffness within 6 months (AE&ROM) (n=923)	
No	801 (86.8%)
Yes	122 (13.2%)
Full rec. defect within 24 months (n=875)	
None	762 (87.1%)
Full recurrent defect	113 (12.9%)
Full rec. defect within 12 months (n=831)	
None	732 (88.1%)
Full recurrent defect	99 (11.9%)

Ipsilateral AE within 12 months (n=973)	
No	690 (70.9%)
Yes	283 (29.1%)
Ipsilateral AE within 24 months (n=973)	
No	659 (67.7%)
Yes	314 (32.3%)
Return to work (n=615)	
mean (sd)	148.2 (115.5)
5th-95th percentile	20.0-392.0

AE: Adverse events; ROM: Range of movement
