# Supplementary material for: Identifying patient-reported and clinician-reported outcome measures to compare providers of arthroscopic rotator cuff repair: insights obtained from a Swiss multicentre cohort study
Source: BMJ Open Qual. 2026 Nov 11;15(4):e004516. doi: 10.1136/bmjoq-2026-004516 (PMC13756451; doi:10.1136/bmjoq-2026-004516)
Supplement: Supplementary file 5 [file bmjoq-15-4-s005.pdf]

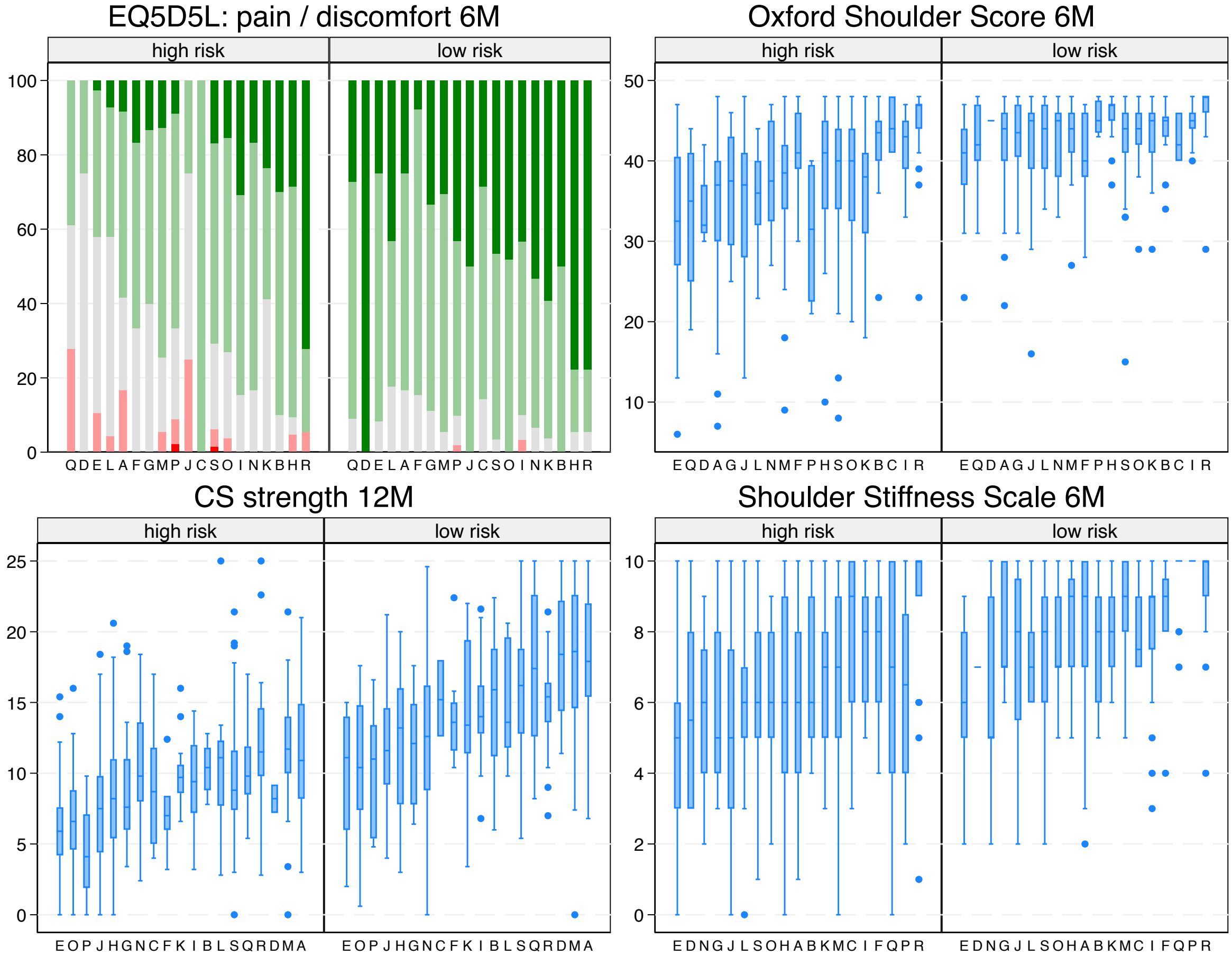

Supplemenetal Figure 1: The distribution of four potential indicators across the 19 clinics stratified by the risk of the patients for an unfavourable outcome. Risk stratification is based on a regression-based predictor of the indicator based on the automatically preselected baseline characteristics and splitting the patients at the median. For all outcomes higher values refer to a more favourable state. In the stacked bar chart, the five possible values are sorted from least favourable (bottom) to most favourable (top). CS: Constant score; 6-month time point.

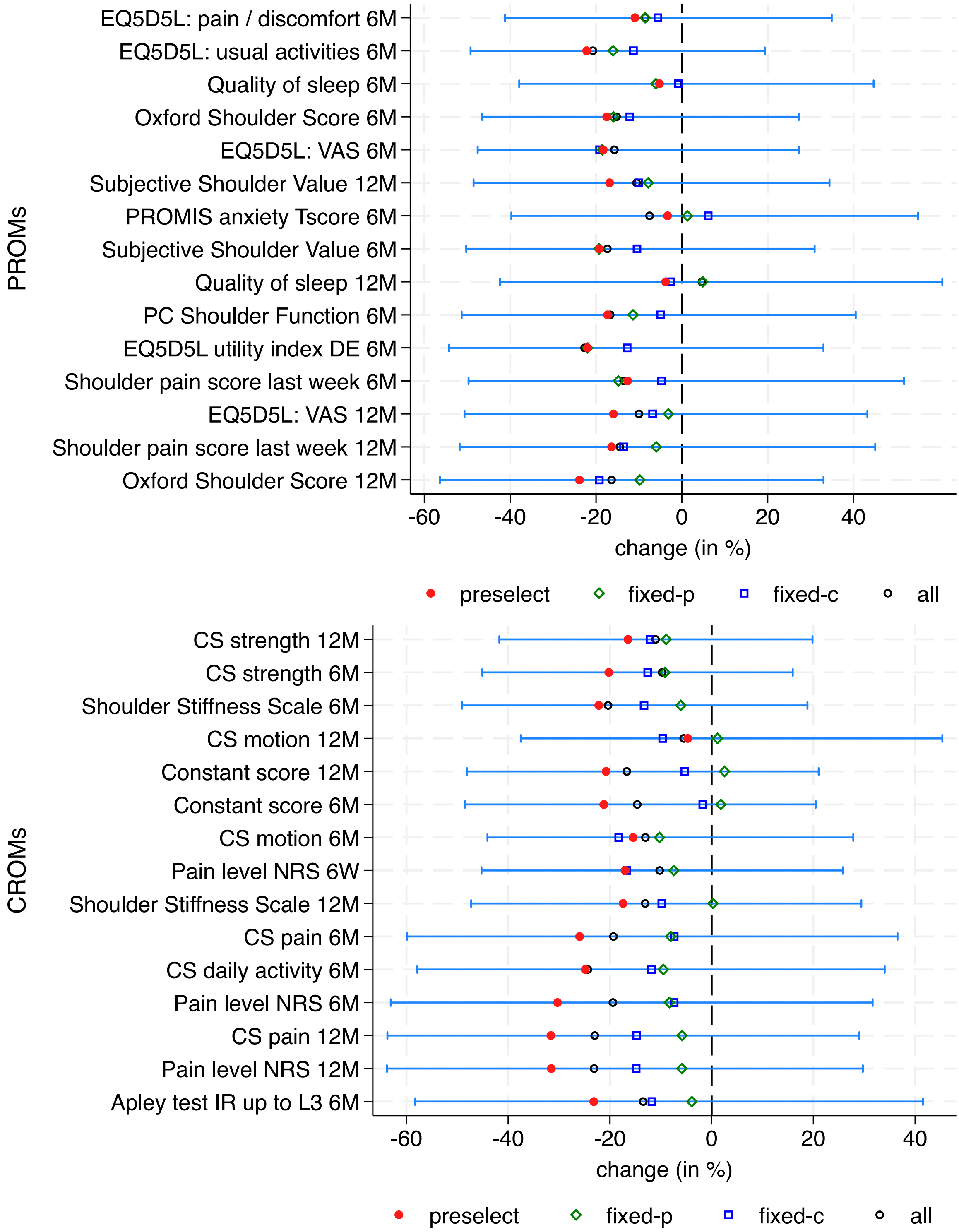

Supplemental Figure 2: The change in clinic variation due to case-mix adjustment compared to no adjustment for the indicators identified in Figure 2. preselect: automated preselection; fixed-p: fixed selection of PROMs; fixed-c: fixed selection of clinical variables; all: all baseline variables. CS: Constant score; IR: Internal rotation; NRS: Numerical rating scale; PC: Perceived change; PROMIS: Patient reported outcomes measurement information system; VAS: Visual analog scale; B: 6W: 6 weeks; 6M: 6 Months; 12M: 12 Months.

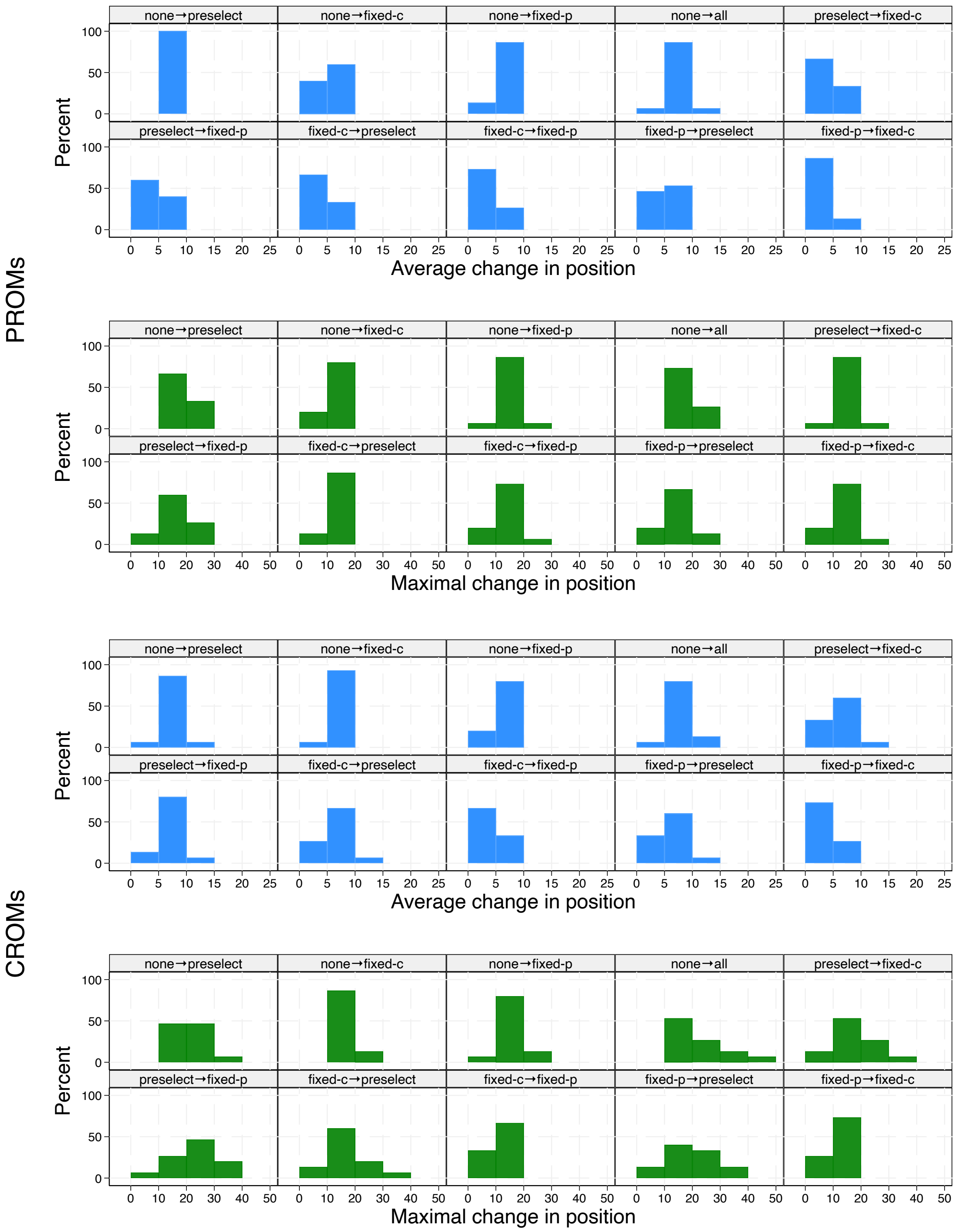

Supplemental Figure 3: The distribution of the average and maximal absolute change in position (across all clinics) when moving from one case-mix adjustment approach to another across the 15 PROMs and CROMs shown in Figure 2. Change in position refers to the absolute change of the standardized mean values expressed as a percentage of the the range of the standardized mean values for the first case-maix adjustment approach. none: no adjustment; preselect: automated preselection; fixed-p: fixed selection of PROMs; fixed-c: fixed selection of clinical variables; all: all baseline variables.
